# Veterinary medicinal product usage among food animal producers and its health implications in Central Ethiopia

**DOI:** 10.1186/s12917-018-1737-0

**Published:** 2018-12-18

**Authors:** Takele Beyene Tufa, Feraol Gurmu, Ashenafi Feyisa Beyi, Henk Hogeveen, Tariku Jibat Beyene, Dinka Ayana, Fanos Tadesse Woldemariyam, Eyerusalem Hailemariam, Fanta Desissa Gutema, J. A. Stegeman

**Affiliations:** 1 College of Veterinary Medicine and Agriculture, Addis Ababa University, P.o. Box 34, Bishoftu, Ethiopia; 2 Department of Farm Animal Health, Faculty of Veterinary Medicine, Utrecht University, Utrecht, The Netherlands; 3 Business Economics Group, Wageningen University & Research, Wageningen, The Netherlands; 4 Center for Outcomes Research and Epidemiology, Department of Diagnostic Medicine and Pathobiology, College of Veterinary Medicine, Kansas State University, Manhattan, KS USA

**Keywords:** Antimicrobial usage, Antimicrobial resistance, Central Ethiopia, Food animals, Farmers’ knowledge, awareness, and practices

## Abstract

**Background:**

Antimicrobials and anthelmintics are the most commonly used veterinary drugs to control animal diseases. However, widespread use of these drugs could contribute to the emergence of drug resistance. Information on the practice of antimicrobial usage among food animal raising communities in Central Ethiopia is scarce. We used a standardised questionnaire survey to assess knowledge, awareness, and practices related to drug use and resistance in food animals among the farmers in and around Bishoftu town.

**Results:**

Of the total of 220 livestock owners interviewed, around 80% of the respondents were not able to define what antimicrobials are and for what purposes they are used. Only 14.1% (*n* = 31) of the respondents had awareness about antimicrobial resistance (AMR) and its consequences; and 35.5% (*n* = 11/31) and 9.7% (n = 3/31) of them agreed that the irrational use of antimicrobials in animals could lead to AMR in animals and humans. Oxytetracycline was the most commonly available antibiotic in veterinary drug shops/pharmacies and the most widely used drug in the area. However, 43.3% of the respondents did not see clinical improvements after using antibiotics. Similarly, the respondents explained that no response was observed in 73.3, 70.8 and 52.5% of the cases after medication with anthelmintics, antiprotozoal and acaricides, respectively. About 56.7% of the respondents considered traditional medicines equally important to modern medicines. It was also noted that there were illegal drug vendors, dispensing medicines under unfavourable conditions which include a direct exposure to sunlight, which practice violates the drug handling and storage recommendations given by WHO.

**Conclusion:**

The study revealed that there is a general lack of awareness among food animal owners about the correct use of antibiotics and anthelmintics. The widespread misuse and improper drug dispensing and handling practices observed in this study can affect the drug quality and can also contribute to the development of drug resistance in central Ethiopia.

## Background

Provision of complete animal health care necessitates the availability of safe, effective and affordable drugs of the required quality in adequate amounts at all times. Veterinary drugs or veterinary medicinal products (VMPs) are necessary to meet the challenges of providing adequate amounts of food for the growing human population, as drugs prevent and cure diseases in food-producing animals [1]. Antibiotics are considered among the most commonly used classes of VMPs for several purposes, including treatment and prevention of infections as well as growth promotion [2]. Antibiotics classified by the WHO as critically important for use in human medicinal care are also used in pigs, poultry, and cattle as growth promoters [3]. Similarly, several anthelmintics are shared between humans and animals, which is a growing problem that is counteracted by annually switching the class of anthelmintics [4].

The use of VMPs, mainly antibiotics, in food animals has raised public health concerns [5] as some of the bacteria in the gastrointestinal tracts are developing resistance to antibiotics [6]. Previous studies have shown that inappropriate uses of antibiotics in animals result in the emergence of antibiotic-resistant microorganisms, which can transfer to humans [7]. Furthermore, infections that were once cured by the introduction of antibiotics are now more difficult to combat because of the emergence of antimicrobial resistant organisms [8]. The irrational and overuse of antibiotics result not only in the development of resistant bacterial strains, but also in adverse health issues such as allergic reactions [2] and economic burdens on the national health system [9, 10].

Indiscriminate use of veterinary drugs, mainly antimicrobials, anthelmintics, and acaricides in food animals also play a major role in the development of antimicrobial resistance (AMR) which has put the public health at risk [11–13]. This problem is further worsened by irrational use through free access to prescription drugs and their administration at sub-therapeutic concentrations for a long period of time [2]. Such conditions favour the selection and spread of antimicrobial resistant strains in animals, environment and humans [14–16].

Studies on the use of antimicrobials and anthelmintics in food animals are of paramount importance to understand the potential risks posed by the emergence of AMR to animals and the public health [11]. Furthermore, understanding the pattern of antimicrobial usage and its occurrence, and identifying associated risk factors will pave a way to taking cost-effective actions [17]. Therefore, this study was designed to assess the knowledge, awareness, and practices of VMPs, focusing on the use of antimicrobials and anthelmintics among food animal owners and animal health professionals.

## Materials and methods

### Study area and design

This study was conducted between December 2013 and March 2014 in central Ethiopia, Bishoftu city and its environs. Farmers around Bishoftu city practice a mixed crop-livestock farming system, where the draft power and the manure of the livestock are used to cultivate the crops, and the crop residues are used as animal feed [18]. Data on the number of animals in the area, number of animals per farm and/or a total number of animals who received treatment by visiting veterinary centers during the study period was not available. Hence, we used a study design where a pre-tested standardised questionnaire was administered by conducting personal interviews with 120 livestock (dairy, beef, and poultry) owners. The livestock owners were selected by means of a stratified random procedure, where from a list of 12 farmers’ associations (locally called ‘Kebele’) surrounding Bishoftu city, six were randomly selected. From each selected Kebele, one village was randomly chosen and 20 food animal owners per Kebele (irrespective of the animal species) were again randomly selected and interviewed. The interviewer contacted heads of the selected households and administered the questionnaire face-to-face in the local language. Additionally, a convenience-based client intercept questionnaire was administered through interviews with 100 animal owners from the villages around Bishoftu city at veterinary clinics and /or pharmacies or drug shops when they came to buy medicines. All (*n* = 9) veterinary pharmacies or drug shops found in Bishoftu city were also visited and inspected for drug storage and arrangement conditions; and the professionals working in them were also interviewed. These interviews were conducted in six farmers’ associations, as well as at the College of Veterinary Medicine and Agriculture (veterinary teaching hospital, the Society for the Protection of Animals Abroad [SPANA] and Donkey Sanctuary projects clinics), at Ada’a district veterinary clinic and nine private veterinary pharmacies. Details of the questionnaire formats administered to the livestock owners and to the professionals working in the veterinary clinics/pharmacies (in the English language) are presented in Annexs 1, 2 and 3.

### Data collection and analysis

For the data collection, we followed the approach described by Gonsalves et al. (2005) [19]. Briefly, the structured questionnaire used for this study included questions related to the pattern of drug use, knowledge assessment about antimicrobials and AMR, the source of the antimicrobial agents, time and method of administration, handling of drugs and general information about the informants such as education level, age, and sex. The gathered data were stored in an excel spreadsheet and analysed using SPSS version 20.

## Results

### Demographics and practice of drug usage among respondents

The majority of the respondents were male (81%, 178/220). Of the 220 respondents, 27% had no formal education, 43% had primary school, 28% secondary school, and 1% had tertiary education. Out of the 100 respondents interviewed at the veterinary clinics and/or pharmacies, 85% visited the clinics to treat (an) animal(s) rather than buy non-prescription medicines, and 77% of them were frequent visitors (more than three times in the last one year).

Among drugs provided to animal owners (*n* = 89/100), anthelmintics were most frequently dispensed (82%). Eighty percent (176/220) of the respondents used only modern medicines, while the remaining 20% also used traditional medicines to treat their animals. Antibiotics and anthelmintics were prescribed to treat different signs and/or symptoms of tentatively diagnosed cases (Table 1). Common signs and symptoms of livestock diseases as described by the owners were associated with the musculoskeletal and integumentary systems (32%), generalised or multiple systems (26%), the digestive system (24%), or the respiratory system (17%). The survey also indicated that livestock owners were purchasing drugs from private drug venders (35.8%), government drug shops (24.2%), or both (40%). In case the animal did not respond to the treatment, 75% of the respondents returned to the same clinic, 14% of them slaughtered or sold the animal, 3% visited a different clinic, 6% switched to traditional medicines while 2% never experienced treatment failure. Sixty-six percent (66/100) of the respondents obtained professional counseling on drug usage from animal-health assistants, while 10% (10/100) and 6% (6/100) obtained this counselling from veterinarians and non-animal health professionals, respectively. However, the remainder (18%) of the respondents did not receive any counseling service. Among animal owners who received professional counseling, 81% of them indicated to have obtained a good understanding of the explanation, whereas 19% did not.

**Table 1:** Antibiotics and anthelmintics prescribed for common clinical signs and/or symptoms of sick animals in and around Bishoftu city, Central Ethiopia

Clinical signs	Anthelmintics		Antibiotics	
	Frequency	Percent	Frequency	Percent
Respiratory*	71	59.2	33	27.5
Musculoskeletal and integumentary**#**	16	13.3	25	20.8
Digestive system**$**	87	72.5	14	11.7
Generalized systemic*#	67	55.8	47	39.2

**Key:** Anthelmintics (albendazole, fenbendazole, tetramisole and ivermectin); Antibiotics (oxytetracycline, penstrep [procaine penicillin and dihydrostreptomycin fixed combination], and sulfa drugs)**;** * Chronic coughing, sneezing, pneumonia and nasal discharge; # Mandibular region swelling, shivering, hernia, trauma and back sore; $ Difficult to defecate, diarrhoea, endoparasites, colic, bloating, no regurgitation, and vomiting; *# Inappetence, salivation, urine discoloration, urination problem, depression, lesion in the mouth, bleeding from mouth and emaciation

Among the 120 farmers interviewed at the six farmers’ associations, 79.2% of them could not read and understand English, and 56.7% of them could not check the expiry date of the drugs. Based on pathognomic clinical signs of livestock diseases, the problems/diseases recently requiring drugs as described by the respondents, were musculoskeletal and integumentary problems (blackleg, ectoparasites, lameness, foot rot, actinomycosis, actinobacillosis, and dermatitis) (74.2%), digestive problems (bloat, endoparasitosis, colic, coccidiosis, and blueish hardened tongue) (59.2%), general systemic problems (anthrax, lumpy skin disease, foot and mouth disease, orf, babesiosis, rabies, uraemia, sheep pox, fowl typhoid, Newcastle disease, and poisoning) (57.5%), respiratory problems (pneumonia, pasteurellosis, and dictyocaulosis) (10%), and reproductive problems (dystocia and abortion) (5%).

The survey also revealed that only 36.7% (81/120) of the respondents practiced deworming of their animals three times a year. The anthelmintic preferences of the farmers showed that 69.2% of them preferred the green colour anthelmintic (albendazole), 16.7% the yellow colour (tetramisole) and the remaining 14.1% did not have any colour preference. When the farmers treated their animals, they determined the dose of anthelmintics by the animals’ age, body condition and body weight (Table 2). Antibiotics commonly used in the study area were oxytetracycline, penstrep (procaine penicillin and dihydrostreptomycin fixed combination), procaine penicillin and sulfa drugs. Though 60.8% of the farmers reported that they did not have information about antibiotics, 11.7% of them used oxytetracycline while the use of other antibiotics was very low. Farmers reported that they could administer antibiotics themselves via different routes (Table 2).

**Table 2:** Ways of dose determination for anthelmintic and route of antibiotics administration by the respondents (*n* = 120) in and around Bishoftu city, Central Ethiopia

Variables	Frequency	Percent
Dose determination for anthelmintics		
Age only	44	36.7
Body condition only	2	1.7
Body weight only	7	5.8
Age and body condition	53	44.2
Age, body condition and body weight	11	9.2
Age and body weight	3	2.5
Routes of antibiotics administration		
Intramuscular	60	50
Oral^a^	57	47.5
Subcutaneous	1	0.8
Don’t know	2	1.7

^a^for chicken

Drugs frequently purchased by the respondents from the veterinary drug stores and pharmacies were antiprotozoal (97.5%), anthelmintics (87.5%), antibiotics (58.3%), and acaricides (1.7%). The respondents mentioned that the anthelmintics were used to treat abnormalities related to the digestive (72.5%), the respiratory (59.2%), generalised (55.8%) and the musculoskeletal and integumentary (13.3%) systems. Similarly, antibiotics treatment was also dispensed for generalised or systemic (39.2%), the respiratory (27.5%), the musculoskeletal (20.8%), and the digestive (11.7%) systems abnormalities (Table 2).

The response of the animal owners regarding the reaction of their sick animals following treatment with antibiotics, anthelmintics, acaricides or antiprotozoal drugs, respectively, indicated that in 43.3, 73.3, 52.5 and 70.8% of the cases, they did not see clinical improvements (Table 3). The survey also showed that most of the farmers (53.4%) used both traditional and modern medicines to treat their animals; however, 40.8% of the farmers used only modern medicines, 3.3% used neither traditional nor modern medicines, and 2.5% used only traditional medicines. Overall, 55.9% of the farmers used specific herbal traditional medicines to treat bloat (32.5%), anthrax (8.3%), blackleg (21.7%) and other disease conditions (10.8%). Most of these traditional medicinal preparations were administered via intra-nasal and oral routes. In general, among the animal owners (*n* = 220), 94.2% of them obtained treatment for their animals by prescription (64%) and non-prescription drugs (30.2%) and the remaining 5.8% never used any modern medicines to treat their animals.

**Table 3:** Response to treatment as complained by respondents in six farmers associations (n = 120 respondents) around Bishoftu, Central Ethiopia

Drug type	Post treatment response of treated animals		
	Good %	No response %	Bad %
Antibiotics	53.3	43.3	3.3
Anthelmintics	25.0	73.3	1.7
Acaricides	46.7	52.5	0.8
Antiprotozoals	29.2	70.8	0.0

Knowledge assessment about the antimicrobials use and resistance revealed that most of the respondents (80%, *n* = 176/220) were not able to define what an antimicrobial is nor the purposes it is used for. Furthermore, only 14.1% (*n* = 31) of the respondents were aware of AMR and its consequences. Among these, only 35.5% (*n* = 11/31) and 9.7% (n = 3/31) of them agreed that the irrational use of antimicrobials in animals could lead to AMR in animals and humans, respectively.

The interviews held with personnel in the veterinary pharmacies/drug shops revealed that anthelmintics (albendazole, ivermectin, tetramisole) and antibiotics (oxytetracycline, penstrep [penicillin and streptomycin fixed combination], and penicillin) were always available in veterinary pharmacies/drug shops. Among these, albendazole and oxytetracycline were the drugs most purchased, followed by acaricides (diazinon and malathion), vitamins (multivitamin) and minerals (calcium borogluconate, ferrous sulphate) (Table 4). The education levels of the personnel working in the veterinary pharmacies/ drug shops were: graduates from high schools (11.1%), from two or three year diploma programme colleges (33.3%) and universities graduates (55.6%). Although all the drug dispensers responded that they were frequently checking the expiry dates of drugs available in their pharmacies/shops, the drug storage and arrangement conditions observed during the visits for the interviews were good (44.4%), fair (33.3%) and poor (22.2%). The criteria for being classified as good, fair, and poor are described in detail in Annex 3.

**Table 4:** Type of drugs available in veterinary pharmacies or drug shops and livestock owners’ preferences in and around Bishoftu, Central Ethiopia

Codes of pharmacies	Type of drugs/ chemicals	
	Drugs available in the shops	Farmers preference to buy by decreasing order
A	AB, AH, anti-inflammatory, local anaesthesia, disinfectants, antidote, antitoxin, acaricides, minerals and vitamins	AB, AH, mineral and vitamins, acaricides, antiprotozoal
B	AB, AH, acaricides, antiprotozoals, minerals and vitamins	AB, AH, acaricides, minerals and vitamins, antiprotozoals,
C	AB, AH, acaricides, antiprotozoal, mineral and vitamin	Minerals and vitamins, AH, AB, acaricides, antiprotozoals
D	AB, AH, acaricides, antiprotozoals, mineral and vitamin	AH, AB, acaricides, minerals and vitamins, and antiprotozoals,
E	AB, AH, disinfectant, acaricides, antiprotozoals, mineral and vitamin	Minerals and vitamins, AH, AB, acaricides, antiprotozoals
F	AB, AH, acaricides, antiprotozoal, minerals and vitamins	AH, AB, acaricides, mineral and vitamin
G	AB, AH, antiprotozoals, minerals and vitamins	AH, antiprotozoals, AB, minerals and vitamins, acaricides
H	AB, AH, acaricides, vitamin and antiprotozoals	AH, antiprotozoals, acaricides, AB, minerals and vitamins
I	AB, AH and vaccines	AH, AB

Key: *AB* antibiotics, *AH* anthelmintics

## Discussion

Inappropriate use of antimicrobials, especially antibiotics shared between humans and animals, plays a leading role in the emergence of AMR [20]. In this survey, the socio-demographic characteristics of the respondents indicated that most of the animal patient encounters were brought to clinics by a male (81%). This may be because culturally most rural women are engaged in the household activities. The survey also revealed that 27 and 43% of the interviewed farmers had no formal education or attended only primary school, respectively. This shows that most of the animal owners (70%) did not understand the information given by drug dispensers or were not able to read and understand the information written on the drug labels/leaflets in English. For this reason, farmers use the colour of drugs to identify them. This study is in line with the study conducted by Regassa et al. (2013) [21].

Diverse groups of antimicrobials were used for all similar clinical signs and symptoms without reaching a final diagnosis. For instances, antibiotics such as oxytetracycline, penstrep, and sulfa drugs were used to treat animals with clinical signs of the respiratory system (27%), the musculoskeletal system (20.8%), the digestive system (11.7%), and signs associated with systemic diseases (39.2%). Misuse of these antibiotics could contribute to the development of antibiotic resistance microorganisms in animals and might facilitate the transmission of resistant bacteria to humans [22, 23]. Similarly, most of the farmers did not use deworming programmes regularly and administered anthelmintics to animals regardless of the animals’ age, body condition and body weight, without prescription from animal health professionals. This may be the reason for a poor response to anthelmintic treatment. This observation has also been noted in the report from Ethiopia by Regassa et al. (2013) [21]. Besides, the drugs’ withdrawal time was not adhered to regarding the use of animal products (milk, meat) for human consumption from the animal under medication. This report is in line with the studies conducted by Beyene et al. (2015) in Ethiopia [24] and by Karimuribo et al. (2005) in Tanzania [25].

Furthermore, studies on the prescribers’ and dispensers’ educational level revealed that the academic qualification of personnel working in the veterinary pharmacies was high school (11%), which may favour the illegal use of the drug owing to their lack of formal training on the handling and dispensing of veterinary medicines. In the study area antimicrobials were sold without prescription papers. Besides, the personnel working in the veterinary pharmacies could also give advice to clients. As these people, however, have no full information about animal diseases, drug use, side effects, withdrawal period and proper route of drug administration, this unprofessional situation can lead to drug residues and/or AMR development. This suggestion is in accordance with the study conducted by Mmbando (2004) [26].

Illegal drug venders found in Bishoftu city did not want to take part in this survey. But observing how they handle the drugs while distributing them to the farmers, showed that they dispensed medicines in a packet in direct sunlight, violating the drug handling and storage recommendations by the WHO [27], and possibly causing substantial changes to the drug active ingredients. On the other hand, legal private drug venders were seen to practice veterinary service just outside of their pharmacy, especially during market days. It was also observed that while the professionals were giving clinical services, a family member stays in the pharmacy and sells the drugs to farmers. Since family members have no knowledge about dispensing drugs, this practice might promote drug misuse that could lead to the development of drug resistance. Poor drug storage conditions further enhance the risk of AMR, which agrees with the conclusions of Komolafe (2003) [28] and Amabile-Cuevas (2010) [29] in Malawi. Furthermore, most animal owners in this study complained about the absence of clinical improvement after their animals had been treated with antimicrobials (antibiotics and antiprotozoals), insecticides or anthelmintics. This could be due to drug misuse (animals receive treatment without a correct diagnosis) or improper dose administrations (which leads to low plasma drug concentration). Both can decrease the clinical effectiveness of the drugs and may also encourage the development of drug resistance. The practices of antimicrobials and other drug used in the study area are not in agreement with the recommendations reported by Le and Munekage (2004) [30].

On the other hand, information collected from the owner on how they manage the diseased animals, revealed high public health risks. For instance, some of the farmers incorrectly used the diseased animal for human consumption or practiced traditional medicines. Some of them consumed the diseased animal meat without proper inspection of the carcass, or without considering the drug withdrawal period. The drug residue available in the edible tissues may cause hypersensitivity/allergic reactions, mutagenicity, and carcinogenicity, in humans, or promote AMR development through a selection of AMR determinants that may spread to human pathogens [1]. Furthermore, 56.7% of the farmers used traditional medicines to treat various animal diseases, whereby most of these traditional preparations were given via the intra-nasal or the oral route. This shows lack of awareness among farmers about the correct use of medicines, the route of administration, and about side effects. These traditionally used medicines themselves may cause a respiratory disease known as drenching pneumonia when they are administered improperly intra-nasally or orally by retracting the tongue of the animal from the buccal area, which is not an uncommon practice.

## Conclusions

This study shows that livestock owners were using similar antimicrobials for all kinds of ailments and that most of them were not aware of the AMR and its consequences. Antibiotics and anthelmintics were misused without proper diagnosis of cases. Drugs were obtained from illegal drug vendors and most livestock owners were illiterate and therefore unable to read and understand the instructions. Mostly, the farmers administered the drugs without getting advice from animal health professionals and by using improper and simple dose estimations based on age and body weight of the animals. This has public health implications as it may lead to failure of medication, development of AMR, and occurrence of drug residues in food animal products. The observed habits of buying drugs based on colours, and using them without proper diagnosis, prescription, dose, frequency, duration, and routes of administration may lead to the emergence of antimicrobial and anthelmintic resistance. Thus, the Ethiopian Veterinary Drug and Feed Administration and Control Authority (VDFACA) should strengthen its surveillance of monitoring illegal drug handlings, and should also raise awareness among livestock owners about rational veterinary drug use and AMR.

## Abbreviations

AMR: Antimicrobial resistance
GIT: Gastrointestinal tract
SPANA: Society for the Protection of Animals Abroad
VTH: Veterinary teaching hospital

## References

[1] Beyene T (2016). Veterinary drug residues in food-animal products: its risk factors and potential effects on public health. J Vet Sci Technol.

[2] Beyene T, Tesega B (2014). Veterinary drug use: its significance in public health. J Vet Med Anim Health.

[3] Wray C, Gnanou J (2000). Antibiotic resistance monitoring in bacteria of animal origin: analysis of national monitoring programs. Int J Antimicrob Agents.

[4] Conly J (2010). Antimicrobial resistance: revisiting the “tragedy of the commons”. Bull World Health Organ..

[5] Collignon P, Wegener HC, Braam P, Butler CD (2005). The routine use of antibiotics to promote animal growth does little to benefit protein under nutrition in the developing world. Food Safety.

[6] Turnidge J, Christiansen K (2005). Antibiotic use and resistance-proving the obvious. Lancet.

[7] Wegener HC. Antibiotic resistance-Linking Human and Animal Health. In: Institute of Medicine (US). Improving Food Safety through a One Health Approach: Workshop Summary. Washington (DC): National Academies Press (US); 2012. A15.http://www.ncbi.nlm.nih.gov/books/NBK114485/. Accessed 22 July 2018. 23230579

[8] Threlfall E, Ward L, Frost J, Willshaw C (2000). The emergence and spread of antibiotic resistance in food-borne bacteria. Int J Food Microbiol.

[9] Gyssens IC (2001). Quality measures of antimicrobial drug use. Int J Antimicrob Agents.

[10] Page SW, Gautier P (2012). Use of antimicrobial agents in livestock. Rev Sci Tech Off Int Epiz.

[11] Landers TF, Cohen B, Wittum TE, Larson EL (2012). A review of antibiotic use in food animals: perspective, policy, and potential. Public Health Rep.

[12] Beyene T, Endalamaw E, Tolossa Y, Feyisa A (2015). Evaluation of rational use of veterinary drugs in Bishoftu, Central Ethiopia. BMC Res Notes..

[13] Magouras I, Carmo LP, Stärk KDC, Schüpbach-Regula G (2017). Antimicrobial usage and -resistance in livestock: where should we focus?. Front Vet Sci.

[14] Zuccato E, Calamari D, Natangelo M, Fanelli R (2000). Presence of therapeutic drugs in the environment. Lancet.

[15] Ethics RB (2001). Science, and antimicrobial resistance. J Agric Environ Ethics.

[16] Thames CH, Pruden A, James RE, Ray PP, Knowlton KF (2012). Excretion of antibiotic resistance genes by dairy calves fed milk replacers with varying doses of antibiotics. Front Microbiol.

[17] Ungemach FR, Müller-Bahrdt D, Abraham G (2006). Guidelines for prudent use of antimicrobials and their implications on antibiotic usage in veterinary medicine. Int J Med Microbiol.

[18] Zeleke A, Bizunesh T, Tesema T. Historical Milestones of Debre Zeit, Agriculture Research center, DARC in half a century (1995-2005). Bulletin of Golden Jubilee. 2005. p.5

[19] Gonsalves J, Becker T, Braun A, Campilan D, De Chavez H, Fajber ME (2005). Participatory Research and Development for sustainable agriculture and natural resource management: a sourcebook. Volume 2: enabling participatory Research and Development.

[20] Hopkins S, Berit Muller-Pebody. UK One Health Report. Joint report on human and animal antibiotic use, sales and resistance, 2013, ed. P. Borriello, M. Sharland. PHE, Wellington House, London; 2015. https://www.gov.uk/government/publications/uk-one-health-report-antibiotics-use-in-humans-and-animals. Accessed 21 July 2018.

[21] Regassa F, Kebede L, Mamo G, Kumsa B, Beyene T (2013). Efficacy of commonly used anthelmintic drugs in naturally infected sheep and goats in Central Oromia, Ethiopia. Res J Pharmacol.

[22] van den Bogaard AE, Stobberingh EE (2000). Epidemiology of resistance to antibiotics links between animals and humans. Int J Antimicrob Agents.

[23] Anderson AD, Nelson JM, Rossiter S, Angulo FJ (2004). Public health consequences of use of antimicrobial agents in food animals in the unites states. Microb Drug Resist.

[24] Beyene T, Kemal A, Jibat T, Tadese F, Ayana D, Feyisa A (2015). Assessment on Chemicals andDrugs Residue in Dairy and Poultry Products in Bishoftu and Modjo, Central Ethiopia. J Nutr Food Sci..

[25] Karimuribo ED, Mdegela RH, Kusiluka LJM, Kambarage DM (2005). Assessment of drug usage and antimicrobial residues in milk on small holder farms in Morogoro, Tanzania. Bull Anim Heal and Prod J.

[26] Mmbando TL (2004). Investigation of OTC used and abuse, Determination of its residues in meat consumed in Dodoma and Morogoro Municipalities. MSc Thesis.

[27] Snow J. Guidelines for the storage of essential medicines and other health commodities. Inc./DELIVER in collaboration with the World Health Organization. 2003. http://apps.who.int/medicinedocs/pdf/s4885e/s4885e.pdf. Accessed 20 Feb 2015.

[28] Komolafe OO (2003). Antibiotic resistance in bacteria- an emerging public health problem. Malawi Med J.

[29] Amábile-Cuevas CF, Sosa A, Byarugaba D, Amábile-Cuevas C, Hsueh PR, Kariuki S, Okeke I (2010). Global perspectives of antibiotic resistance. Antimicrobial resistance in developing countries.

[30] Le TX, Munekage Y (2004). Residues of selected antibiotics in water and mud from shrimp ponds in mangrove areas, in Vietnam. Mar Pollut Bull.

